# Prioritising topics for developing e-learning resources in healthcare curricula: A comparison between students and educators using a modified Delphi survey

**DOI:** 10.1371/journal.pone.0253471

**Published:** 2021-06-24

**Authors:** Hooi Min Lim, Chirk Jenn Ng, Chin Hai Teo, Ping Yein Lee, Puteri Shanaz Jahn Kassim, Nurul Amelina Nasharuddin, Phelim Voon Chen Yong, Renukha Sellappans, Wei Hsum Yap, Yew Kong Lee, Zahiruddin Fitri Abu Hassan, Kuhan Krishnan, Sazlina Shariff Ghazali, Faridah Idris, Nurhanim Hassan, Enna Ayub, Stathis Konstantinidis, Michael Taylor, Cherry Poussa, Klas Karlgren, Natalia Stathakarou, Petter Mordt, Arne Thomas Nilsen, Heather Wharrad

**Affiliations:** 1 Faculty of Medicine, Department of Primary Care Medicine, University Malaya, Kuala Lumpur, Malaysia; 2 Faculty of Medicine and Health Sciences, Department of Family Medicine, Universiti Putra Malaysia, Serdang, Malaysia; 3 Faculty of Computer Science and Information Technology, Department of Multimedia, Universiti Putra Malaysia, Serdang, Malaysia; 4 Faculty of Health & Medical Sciences, School of Biosciences, Taylor’s University, Subang Jaya, Selangor, Malaysia; 5 Faculty of Health & Medical Sciences, School of Pharmacy, Taylor’s University, Subang Jaya, Selangor, Malaysia; 6 Faculty of Built Environment, Department of Building Surveying, University Malaya, Kuala Lumpur, Malaysia; 7 Faculty of Medicine, Medical Research and Development Unit, University Malaya, Kuala Lumpur, Malaysia; 8 Faculty of Medicine and Health Sciences, Department of Pathology, Universiti Putra Malaysia, Serdang, Malaysia; 9 E-Learning Academy, INTELLECT, Taylor’s University, Subang Jaya, Selangor, Malaysia; 10 School of Health Sciences, University of Nottingham, Nottingham, England; 11 Department of Learning, Informatics, Management and Ethics (LIME), Karolinska Institutet, Solna, Sweden; 12 NettOp, Department of E-Learning Development, University of Stavanger, Stavanger, Norway; Endeavour College of Natural Health, AUSTRALIA

## Abstract

**Background:**

Engaging students in the e-learning development process enhances the effective implementation of e-learning, however, students’ priority on the topics for e-learning may differ from that of the educators. This study aims to compare the differences between the students and their educators in prioritising the topics in three healthcare curricula for reusable e-learning object (RLO) development.

**Method:**

A modified Delphi study was conducted among students and educators from University Malaya (UM), Universiti Putra Malaysia (UPM) and Taylor’s University (TU) on three undergraduate programmes. In Round 1, participants were asked to select the topics from the respective syllabi to be developed into RLOs. Priority ranking was determined by using frequencies and proportions. The first quartile of the prioritised topics was included in Round 2 survey, which the participants were asked to rate the level of priority of each topic using a 5-point Likert scale. The mean score of the topics was compared between students and educators.

**Result:**

A total of 43 educators and 377 students participated in this study. For UM and TU Pharmacy, there was a mismatch in the prioritised topics between the students and educators. For UPM, both the educators and students have prioritised the same topics in both rounds. To harmonise the prioritisation of topics between students and educators for UM and TU Pharmacy, the topics with a higher mean score by both the students and educators were prioritised.

**Conclusion:**

The mismatch in prioritised topics between students and educators uncovered factors that might influence the prioritisation process. This study highlighted the importance of conducting needs assessment at the beginning of eLearning resources development.

## Introduction

Conventionally, in healthcare education, various learning methods have been used; these include lecture-based learning, the Socratic method with the use of questioning and cross-examining, case-based learning and problem-based learning [1]. In recent years, different modalities of e-learning have increasingly been used in healthcare education. The main advantages of e-learning are its flexibility and accessibility where learners can learn at their own pace wherever they are. E-learning in healthcare education has been shown to effectively enhance learners’ understanding of difficult topics or concepts with the use of technology [2].

Traditionally, practical skills learning in healthcare curriculum were deemed to be not suitable for e-learning [3]. Certain types of training in healthcare such as interpersonal skills and communication skills might be considered to be less appropriate for e-learning delivery [4]. With the introduction of the use of stimulation technology, virtual patients and synchronous learning delivery, more e-learning materials on practical skills and soft skills have been developed in the healthcare curriculum [5]. The use of blended learning has overcome some of the disadvantages of e-learning by integrating face-to-face teaching with online e-learning materials [6,7]. However, there is a lack of literature and guidelines to determine suitable topics that can be effectively implemented with e-learning in the healthcare curriculum.

Engagement of students in medical education especially student participation in curriculum development is getting more attention in recent years [8–10]. Students as co-creators in the medical curriculum provide input from the learners’ perspective, continuous feedback and innovations to improve the curriculum [8,9,11]. In e-learning development, learning needs assessment among the students is the initial step to identify the needs and preferences of the end-users [12]. It is important to assess students’ needs, views and preferences in e-learning as these are important aspects for effective implementation and integration of e-learning into the curriculum. Students can give valuable input on the topics they are struggling to understand that e-learning might be helpful or which topics can be supplemented with e-learning to enhance their learning experience. However, students and educators might have different opinions on the types of curriculum that can be effectively supplemented with e-learning. To date, there is no study on how to identify the topics for e-learning object development in the healthcare curriculum.

Identifying needs and topics for RLO development in the existing curriculum is the first step in the development process [13,14]. This study to compare the differences between the students and their educators in prioritising the topics for RLO development.

## Materials and methods

This study was part of the Advancing Co-creation of RLOs to Digitise Healthcare Curricula (ACoRD) project, which is an Erasmus Plus funded 3-year project with the collaboration of six institutions from Europe and Malaysia. This project aimed to introduce innovative digital pedagogy methods that will benefit the healthcare and biomedical science students in partner countries. Reusable learning objects (RLOs), which are small 10–15 minutes of interactive e-learning objects which focus on a single learning goal, will be developed in this project.

### The modified Delphi survey

A two-round modified Delphi survey was conducted from January to March 2019 in three different universities in Malaysia, i.e. University of Malaya (UM), Universiti Putra Malaysia (UPM) and Taylor’s University (TU) to identify the prioritised topics for RLO development. Table 1 shows the healthcare curricula selected for the development of RLOs. Each institution selected the specific curriculum for which the RLO development will be integrated into, based on the institution’s priority and researchers’ interests. For TU, two courses (Pharmacy and Biomedical) participated in this modified Delphi survey. As the response rate from TU Biomedical was low, where only one educator responded in Round 2 (Round 1: 3 educators, 48 students; Round 2: 1 educator, 40 students), we decided to exclude TU Biomedical from the results and only reported the findings from TU Pharmacy (S1 Table).

**Table 1:** Healthcare curricula selected for development of reusable learning objects (RLOs).

Institutions	Programme	Curriculum	Number of topics for selection
University of Malaya (UM)	Undergraduate Medical Programme (Undergraduate Year 4–5)	Primary Care Medicine	84
Universiti Putra Malaysia (UPM)	Undergraduate Medical programme (Year 1–5)	Personal and Professional Development	34
Taylor’s University (TU Pharmacy)	Pharmacy undergraduate Programme (Year 1–2)	Microbiology, Biochemistry, Anatomy & Physiology	87

The Delphi technique uses systematically repeated rounds of iterative questionnaire exercise with controlled feedback to achieve expert consensus [15,16]. The Delphi method is commonly used in healthcare education to achieve consensus over curricular needs or to set priorities [15,17–19]. It also has been used in different medical specialties to identify and prioritise the topics and procedures for training and teaching in medical education [20–22]. In the original Classical Delphi process, round 1 is usually an open-ended questionnaire asking the panelists for their opinions on a certain issue for idea generation. These responses are then analysed by the researchers and feedbacked to the panellists for Round 2 in the form of statements or questions for rating or ranking. In subsequent rounds, the panellists are provided with the responses from other participants and are asked to reconsider their responses. The rounds continue until a consensus is reached [23].

**Fig 1:**
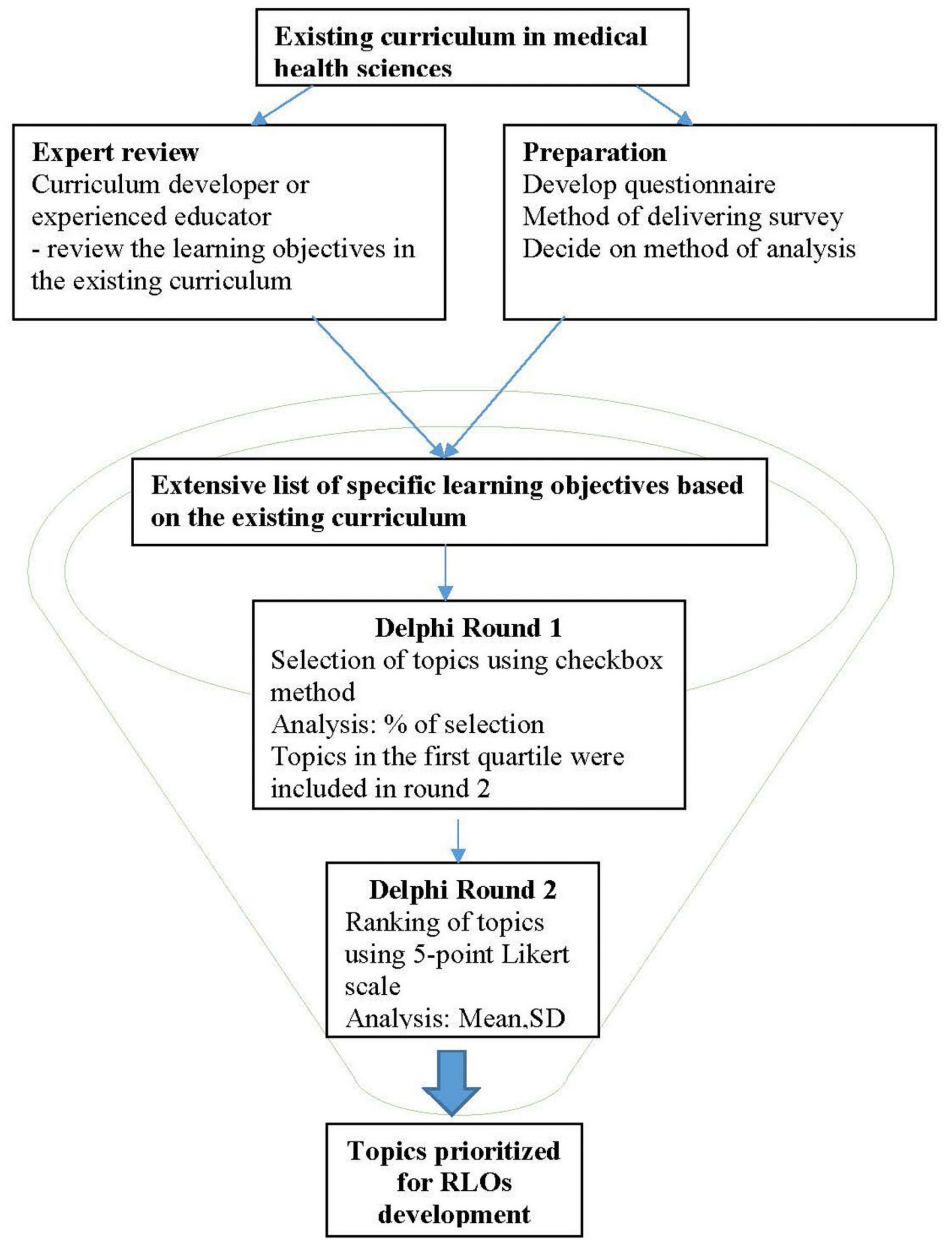
Flowchart of the modified Delphi survey in prioritising topics for reusable learning object (RLO) development, a funnel decision-making model.

In this study, the conventional first round of a Delphi study was omitted because the list of topics in the questionnaire can be identified from the existing curricula of the respective institutions. Fig 1 shows the flowchart of how the topics were prioritised using the modified Delphi survey. An expert panel consisting of the curriculum developers and experienced educators reviewed the learning objectives in the existing curriculum. The expert identified a specific subject area or a course. They reviewed the learning outcomes/topics within a course and listed them into a questionnaire for Round 1.

### Participants

In Round 1, the participants in this study were (1) students from the respective programs who have completed the specific course and (2) educators who were currently teaching in the course. In Round 2, participants are those who responded to the Round 1 questionnaire.

### Data collection and analysis

The research team identified the participants from the respective department and invited them individually. A universal sampling method was used, and all students who have completed the specific course and educators involved in teaching the specific course were invited. An online survey was conducted using RedCap (UM) and Google Form (UPM and TU). The participants read through the participant information sheet and signed the online consent form if they agreed to participate. Subsequently, the participants filled up the demographic data and the main questionnaire. The study was approved by the University of Malaya Medical Centre Medical Research Ethics Committee (MECID No 2019225–7166) and The Ethics Committee for Research Involving Human Subjects Universiti Putra Malaya (JKEUPM-2019-103).

### Round 1

In Round 1, all the eligible participants received an invitation via email or WhatsApp message, and reminders were sent two weeks later. First, participants were explained on the concept and characteristics of RLOs. They were asked to select the topics that need to be supplemented with RLOs, either topic which needs RLOs to underline their importance or more complex topics where RLOs are needed to enhance students’ understanding. The participants were provided with a comprehensive list of topics and they selected the topics using a checkbox method. Within each institution, the data were analysed separately according to the student and educator categories, and the percentage was calculated for each topic (number of participants who checked on a topic divided by a total number of participants). The topics were sorted in descending order (the most selected to least selected topics) separately for student and educator groups. The first quartile of most selected topics from the students and the first quartile of most selected topics from educators were included in the Round 2 survey.

### Round 2

Participation in the previous Round 1 was a requirement for participation in the Round 2 survey. In Round 2, the participants were not aware whether the topic was chosen by the student or educator to avoid bias. Subsequently, the participants were asked to prioritise each of the topic listed to be developed into an e-learning object using a 5-point Likert scale (1-not a priority, 2-low priority, 3-medium priority, 4-high priority, 5-essential). The mean and standard deviations of each topic were calculated and sorted in descending order. Scatter plot was used to compare the topic prioritisation of students in Round 1 and Round 2. It was also used to compare the difference of topic prioritisation between students and educators. All the data were analysed using SPSS version 21.

## Results

Table 2 shows the demography of the participants in this study. A total of 43 educators and 377 students from the three institutions participated in this study. The response rate for Round 1 was 69.7% (UM 71.7%, UPM 56.1%, TU Pharmacy 81.2%) and round 2 was 72.3% (UM 68.7%, UPM 64.3%, TU Pharmacy 83.9%) (S1 Table).

**Table 2:** Demography of participants (n = 420).

**Educators**		**UM (n = 15)**	**UPM (n = 25)**	**TU (Pharmacy) (n = 3)**
Gender (n, %)	Male	4 (26.7)	6 (24)	1 (33.3)
	Female	11 (73.3)	19 (76)	2 (66.7)
Academic qualification (n, %)	Master	9 (60)	12 (48)	0
	PhD	6 (40)	13 (52)	3 (100)
Years of teaching (median, IQR)		9 (13)	8 (5)	2.5 (3.5)
Hours spent in teaching the specified curriculum per week (mean±SD)		4.4±3.6	7.5±10.9	8.7±3.4
**Students**		**UM (n = 119)**	**UPM (n = 205)**	**TU (Pharmacy) (n = 53)**
Gender (n, %)	Male	56 (47.1)	56 (27)	14 (26.4)
	Female	63 (52.9)	149 (73)	39 (73.6)
Current year of study (n, %)	Year 1	-	-	29 (54.7)
	Year 2	-	14 (7)	24 (45.3)
	Year 3	-	67 (33)	-
	Year 4	34 (28.6)	76 (37)	-
	Year 5	85 (71.4)	48 (23)	-

*UM University Malaya; UPM Universiti Putra Malaysia; TU Taylor’s University; IQR interquartile range; SD standard deviation.

**Fig 2:**
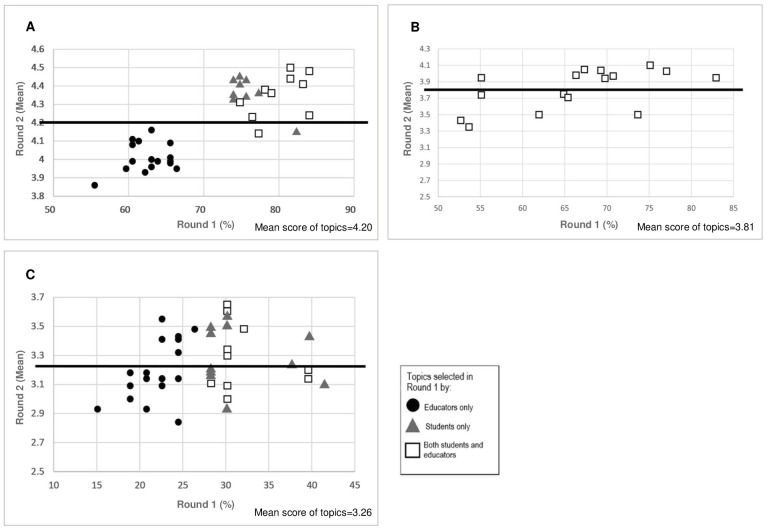
Comparison of topic prioritisation in Round 1 (percentage of selection) and Round 2 (mean score) by the students and educators. (A) University of Malaya (B) Universiti Putra Malaysia (C) Taylor’s University. Only the first quartile of most selected topics by each party (students or educators) in Round 1 were plotted in the scatter plot. The line represents the mean score of the topics in Round 2.

Fig 2 shows the comparison of topic prioritisation between Round 1 (percentage of selection) and Round 2 (mean score of each topic) by the students and educators. For UM in Round 1, there was a difference in the topics selected by the students and educators. In Round 2, the students gave a higher mean score (Round 2) to the topics selected by themselves compared to those selected only by the educators (Fig 2A). For UPM, on the other hand, both the students and educators selected the same topics in Round 1 (Fig 2B). Hence, there was no discrepancy in the topic selection between students and educators in Round 2 survey. For TU Pharmacy, none of the topics reached a consensus of ≥ 50% from the students in Round 1 (Fig 2C). In Round 2, students gave a higher score on six topics that were only selected by the educators on Round 1. Eleven topics selected by students in Round 1 had a lower mean score in Round 2. There was inconsistency with the selection of the topic in Round 1 and Round 2 by the students.

**Fig 3:**
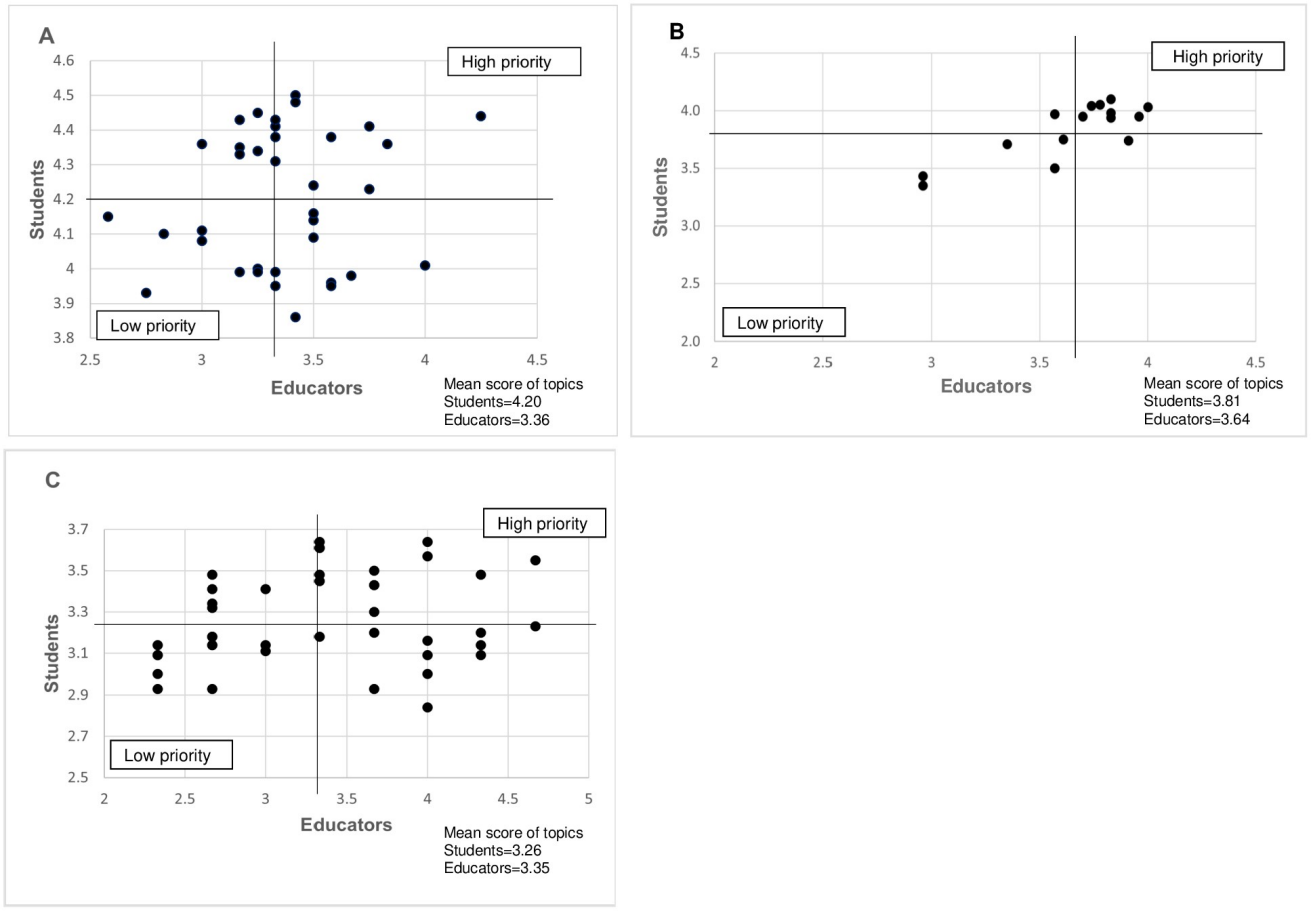
Comparison of the mean score of each topic given by the students and educators in Round 2. (A) University of Malaya (B) Universiti Putra Malaysia (C) Taylor’s University. A scatter plot was used to harmonise the topics prioritisation between students and educators by comparing the mean score of each topic in Round 2 (X-axis is the mean score of each topic by educators, Y-axis is the mean score of each topic by students). The lines represent the mean score of the topics.

Fig 3 shows the comparison of mean scores for each topic between the students and educators in Round 2. To harmonise the prioritisation of topics between students and educators, the topics with higher scores by both the students and educators will be selected to be developed into RLOs. For UM, the topics with mean score ≥ 3.36 by the educators and mean score ≥ 4.20 by the students will be prioritised for RLO development (Fig 3A). For UPM, the prioritised topics were consistent between the students and educators (Fig 3B). For TU Pharmacy, the topics with mean score ≥ 3.35 by the educators and mean score ≥ 3.26 by the students will be prioritised for e-learning development (Fig 3C). For UM and TU Pharmacy, the topics with a lower mean score by both the educators and students would not be prioritised for e-learning development.

## Discussion

This study demonstrated a great variation on how students prioritised topics for RLO development in each institution. For UPM, students and educators prioritised the same topics for RLO development. For UM and TU Pharmacy, there was a mismatch in the prioritised topics between the students and educators. Students from UM were consistent with their choice of topics in both Round 1 and Round 2, however, students from TU Pharmacy were not consistent with the choice of topics in Round 2 as compared to Round 1. To harmonise the prioritisation between the students and educators in UM and TU Pharmacy, the topics with higher mean scores by both the students and educators would be selected for RLO development.

The students and educators in UPM were congruent in their selection of topics for RLO development. This could be because, the personal and professional development curriculum in UPM has fewer topics (n = 34) as compared to UM (n = 84) and TU Pharm (n = 87). The scope of the personal and professional curriculum was more focused, aiming at professionalism, ethics and law, cultural competence, and evidence-based medicine [24,25].

For UM, there was a clear discrepancy in the topics selected by the students and educators for RLO development. This could be due to the scope of the primary care medicine curriculum in UM was wide, ranging from knowledge and comprehension of principles to specific clinical skills such as communication and procedural skills. Misconceptions of the practice and principles of primary care medicine among students might be contributing to the mismatch in topics between students and educators [26–28]. A study reported that students focused on the psychosocial and human aspects of primary care medicine but less emphasized on the technical aspects such as managing uncertainty and clinical reasoning of primary care practice [27]. Some of the students appeared to focus on the organ or disease-based medical knowledge instead of holistic and patient-centred approach in primary care medicine [26]. More exploration needed to determine the reasons for a discrepancy, whether caused by the presence of mismatch teaching and learning focus between students and educators, or different opinions between students and educators on the types of topics that should be supplemented with RLOs in the primary care medicine curriculum.

For TU Pharmacy with the curriculum on basic science topics (microbiology, biochemistry, anatomy and physiology), students prioritised the educators’ choice of topics over theirs. With the use of the Delphi survey where the choices are anonymous, students have a chance to reconsider the priority of topics in the subsequent round, unconsciously reconsidered the topics which were prioritised by the educators in the previous round. It is possible that the students’ views could have changed because some students were less confident and changed to the majority viewpoint [29]. However, this pattern was not observed in the group of UM students where the students remained consistent with the topics of their choice in Round 2, not influenced by the educators’ topics. Another possible explanation for differences in prioritisation pattern across different universities is the nature of the topics. For instance, TU’s topics were mainly on basic science (knowledge-based) while those of UM and UPM focused on clinical competencies (e.g. prescription and communication). In addition, TU had a large number of topics for selection but only a smaller number of participants, especially educators. This again might contribute to the difference in the prioritisation process for TU.

In this present study of prioritising topics for RLO development, different results are expected when the same methodology is carried out in different institutions because of the variation in the existing curriculum, regional needs, teaching methods, learning activities and pace of e-learning development in each institution. More research, especially using a qualitative approach, is needed to explore why students select topics differently from their educators, how the students and educators select the topics for RLOs and what factors they are considering when they prioritise. Understanding these will help to formulate a better methodology in prioritising topics for RLO development.

In the current higher education teaching processes, the student-centred approach has been implemented as the students in higher education have the maturity to understand the required standard and learning objectives [30]. Often, students have a higher expectation in their learning processes causing a mismatch between the educators’ perception and students’ expectation [31–33]. Lack of knowledge and understanding among the faculty and educators could be a factor that contributed to an unmet expectation among the students. Hence, it is important to take the students’ preference of topics into consideration when developing RLOs. Students have their own experiences what topics that are difficult to understand where RLO would be helpful; or what topics require more multimedia interaction to enhance their learning processes. However, educators’ opinions might be subjected to their personal interest, subspeciality or perception. While educators’ opinions on the selection of topics for RLO development should be considered as they are the content and education experts, their preference and prioritisation may be influenced by their personal interest and perceptions of the students. Afshar et al. [34] have highlighted how educator’s preferred teaching approach resulted in the loss of interest and reluctance in learning biochemistry among medical students, and how this can be addressed by taking into considerations of the students’ learning needs. We would, therefore, like to argue that teaching approaches should prioritise students’ needs and preferences over those of the educators. In addition, students’ need assessment should be the cornerstone in curriculum planning and development to allow educators in identifying topics, skills and knowledge that address learning needs [35]. With needs assessment, learning becomes more relevant to their clinical practice, which is more likely to lead to a change in their practice [36].

For the final topic selection for RLO development, topics with a higher priority by both students and educators would be chosen because these topics meet the learning or teaching needs of both parties. We would expect the RLOs to effectively improve the learning outcomes and have a higher success rate of implementation if the topics were chosen by both end-users of the RLOs. Likewise, topics with low priority by both the students and educators would not be prioritised for RLO development. For the topics which were only prioritised by one party, either students or educators only, more stakeholders’ input is needed to decide on the necessity of these topics to be developed into RLOs. Harmonising the topics prioritisation between students and educators is important to balance the opinions of both parties and carefully choose the topics for RLO development especially when the resources for e-learning development are limited and high-quality multimedia content is expensive to develop.

This present study demonstrated a practical approach using a modified Delphi survey to prioritise the topics systematically for RLO development. It allows the anonymity and confidentiality of the participants and students can prioritise the topics independently and objectively, free from peer pressure and educators’ dominance. The data in this study were analysed separately between the students and educators, so the opinions from both parties would be considered. Analysing both parties together would diminish the opinions of the educators as students often outnumbered educators in an institution. The modified Delphi survey is one of the methods that engages the students for RLO development in the curriculum. This present study was not aimed to achieve a consensus but rather described on the practical approach used to prioritise the topics which is applicable for different disciplines in healthcare curricula. This present study offered a basis for embedding the concept of topic prioritisation to develop highly relevant and useful eLearning objects. Engaging the end-users in topic selection is important for the effective implementation of RLOs.

The strength of this study was that it was conducted in three higher education institutions in Malaysia, with input from both students and educators as stakeholders. This study showed the feasibility of conducting a modified Delphi survey across different institutions and healthcare curricula. This was also the first study examining the variation in the selection of topics for RLO development among institutions. However, there are some limitations to this study. Firstly, the majority of topics selected by the students from TU Pharmacy did not achieve >50% of consensus in Round 1, which indicated low agreement of the topics. Second, there were smaller number of educators in this study especially in TU which may not reflect the ‘true’ needs of the educators involved in the teaching of the curricula. The nature of topics was different between institutions where UM’s and UPM’s topics were related to clinical competencies while TU Pham’s topics were basic science knowledge. This might contribute to the discrepancy of results between institutions. The students in this study were at different stages of their undergraduate programmes; this might have affected their learning needs and experience. This factor was not further explored in this study.

## Conclusions

This study showed the variation of opinions in topic selection between students and educators across institutions and health topics. Further research is needed to explore the factors influencing the discrepancy in students’ learning needs from the perspective of the students and educators. This study also highlighted the importance of conducting students’ learning needs assessment before developing eLearning resources for effective implementation. Learning needs assessment should be the starting point when designing eLearning resources for healthcare curricula.

## Supporting information

S1 Table Response rate of the modified Delphi survey according to institutions. (PDF)

S2 Table Percentage of selection in Round 1 and mean score in Round 2 for each topic. (PDF)
