# Supplementary material for: Prioritising topics for developing e-learning resources in healthcare curricula: A comparison between students and educators using a modified Delphi survey
Source: PLoS One. 2021 Jun 24;16(6):e0253471. doi: 10.1371/journal.pone.0253471 (PMC8224897; doi:10.1371/journal.pone.0253471)
Supplement: S1 Table — (PDF) [file pone.0253471.s001.pdf]

S1 Table. Response rate of the modified Delphi survey according to institutions.

	Institutions	UM		UPM		TU (Pharmacy)		TU (Biomed)	
		Round 1	Round 2	Round 1	Round 2	Round 1	Round 2	Round 1	Round 2
Educators	Response rate (%) (Number responded/ number of educators)	78.9 (15/19)	80 (12/15)	83.3 (25/30)	80 (20/25)	100 (3/3)	100 (3/3)	50 (3/6)	33.3 (1/3)
Students	Response rate (%) (Number responded/ number of students)	70.8 (119/168)	67.2 (80/119)	53.9 (205/380)	61.9 (127/205)	80.3 (53/66)	83 (44/53)	85.7 (48/56)	83.3 (40/48)
Total Response	Total Response rate (%) (Total number responded/ total number of students and educators)	71.7 (134/187)	68.7 (92/134)	56.1 (230/410)	64.3 (148/230)	81.2 (56/69)	83.9 (47/56)	82.3 (51/62)	80.4 (41/51)
								TU (Biomed) was excluded from this manuscript because there was only one educator responded in Round 2.	

UM University Malaya; UPM University Putra Malaysia; TU Taylor's University; Biomed biomedical
