# Supplementary material for: Prioritising topics for developing e-learning resources in healthcare curricula: A comparison between students and educators using a modified Delphi survey
Source: PLoS One. 2021 Jun 24;16(6):e0253471. doi: 10.1371/journal.pone.0253471 (PMC8224897; doi:10.1371/journal.pone.0253471)
Supplement: S2 Table — (PDF) [file pone.0253471.s002.pdf]

S2 Table. Percentage of selection in Round 1 and mean score in Round 2 for each topic

S2(A) Table: University Malaya

		Round 1				Round 2	
		Educators		students		Educators	Students
	Topic	n	%	n	%	Mean±SD	Mean±SD
1	Principles of primary care medicine	10	66.67	78	65.55	4.0±1.1	4.01±0.95
2	Applying the knowledge of basic clinical science in primary care medicine and recall transferable knowledge	3	20.00	98	82.35	2.58±1.1	4.15±0.86
3	Principles of diagnostic and therapeutic procedures	9	60.00	100	84.03	3.5±1.2	4.24±0.80
4	Recognizing how culture and beliefs can have an impact on patient	7	46.67	68	57.14	-	-
5	Role of primary care physicians and other team members	8	53.33	66	55.46	3.42±1.0	3.86±0.87
6	Medico-legal and ethical issues in primary care practice	8	53.33	92	77.31	3.50±0.9	4.14±0.82
7	Gathering and analyzing relevant clinical information from history and physical examination for diagnostic and management purposes	9	60.00	93	78.15	3.33±1.0	4.38±0.66
8	Identifying and selecting appropriate investigations for: Diagnostic purposes (referring to patient's safety, specificity and sensitivity of each investigation, cost-effectiveness)	9	60.00	99	83.19	3.75±0.62	4.41±0.65
9	Identifying and selecting appropriate investigations for: Monitoring progression of a pathology	7	46.67	92	77.31	3.00±0.74	4.36±0.62
10	Identifying and selecting appropriate investigations for: Treatment response	7	46.67	90	75.63	3.25±0.75	4.34±0.67
11	Treatment plans for the patient: Short term	11	73.33	97	81.51	3.42±0.79	4.5±0.66
12	Treatment plans for the patient: Long term	11	73.33	100	84.03	3.42±0.79	4.48±0.66
13	Principles of safe and rational prescribing: Prescribing and counselling on medication use	11	73.33	97	81.51	4.25±0.45	4.44±0.67
14	Principles of safe and rational prescribing: Practicing prescription writing	11	73.33	94	78.99	3.83±1.0	4.36±0.77
15	Principles of safe and rational prescribing: Counselling on the use of therapeutic medical devices for the delivery of medication	11	73.33	91	76.47	3.75±1.0	4.23±0.73

16	Performing an accurate and focused history, relevant clinical examination, differential diagnosis and initial management plan (investigation, prescribing medications and counselling) for common presenting complaints in primary care	7	46.67	90	75.63	3.17±0.72	4.43±0.69
17	Abdominal pain	7	46.67	89	74.79	3.25±0.75	4.45±0.59
18	Back pain	7	46.67	87	73.11	-	-
19	Chest pain	7	46.67	86	72.27	-	-
20	Constipation	7	46.67	90	75.63	3.25±0.75	4.34±0.76
21	Cough	7	46.67	88	73.95	3.17±0.72	4.35±0.70
22	Diarrhoea	7	46.67	83	69.75	-	-
23	Dizziness	7	46.67	85	71.43	-	-
24	Dyspnoea	7	46.67	84	70.59	-	-
25	Eye redness	7	46.67	89	74.79	3.33±0.89	4.41±0.69
26	Fever	6	40.00	88	73.95	3.33±0.89	4.43±0.63
27	Headache	7	46.67	84	70.59	-	-
28	Joint pain	7	46.67	84	70.59	-	-
29	Leg swelling	7	46.67	80	67.23	-	-
30	Loss of weight	6	40.00	85	71.43	-	-
31	Nausea and vomiting	7	46.67	84	70.59	-	-
32	Painful ear	7	46.67	84	70.59	-	-
33	Palpitation	7	46.67	88	73.95	3.17±0.72	4.33±0.71
34	Skin rash	7	46.67	82	68.91	-	-
35	Tiredness	7	46.67	87	73.11	-	-
36	Management of dying, death and bereavement in the community setting	5	33.33	82	68.91	-	-
37	Identifying and evaluating the best available medical evidence for use in clinical decision making:<br>Search engines	10	66.67	75	63.03	3.58±0.52	3.96±0.88
38	Pre-appraised evidence	10	66.67	71	59.66	3.33±0.65	3.95±0.86
39	Clinical pathways	9	60.00	79	66.39	3.58±0.67	3.95±0.81
40	Clinical guidelines	9	60.00	93	78.15	3.58±0.79	4.38±0.70

41	Practice protocols	9	60.00	89	74.79	3.33±0.65	4.31±0.69
42	Communicating with:<br>Patients and relatives	9	60.00	75	63.03	3.25±1.14	4.00±0.97
43	Colleagues from a range of health and other professions effectively	9	60.00	72	60.50	3.17±0.84	3.99±0.98
44	Communicating skillfully with patients and their significant others:<br>exhibiting empathy	6	40.00	66	55.46	-	-
45	establishing rapport	6	40.00	66	55.46	-	-
46	engendering confidence and trust	6	40.00	65	54.62	-	-
47	valuing patient autonomy	6	40.00	64	53.78	-	-
48	valuing patient privacy and confidentiality	6	40.00	67	56.30	-	-
49	Using communication styles appropriate for the context (social and cultural) of the consultation	7	46.67	64	53.78	-	-
50	Using verbal and non-verbal communication styles for various situations:<br>Difficult consultation	8	53.33	78	65.55	3.25±1.14	3.99±1.00
51	Breaking bad news	8	53.33	78	65.55	3.50±1.1	4.09±0.93
52	Taking sexual history	8	53.33	78	65.55	3.67±1.2	3.98±0.89
53	Dealing with angry patients	8	53.33	76	63.87	3.33±1.1	3.99±0.89
54	Advising patients with regard to investigative and treatment options, incorporating shared decision making	8	53.33	72	60.50	3.00±0.95	4.11±0.84
55	Providing relevant health information and education to patients and families	6	40.00	77	64.71	-	-
56	Acquiring clinical presentation skills appropriate to the context	5	33.33	68	57.14	-	-
57	Counselling patients on lifestyle modification:<br>Principles of counselling	7	46.67	74	62.18	-	-
58	Prochaska: Stages of change	7	46.67	74	62.18	-	-
59	Counselling on lifestyle modification for diet, exercise, smoking and obesity	8	53.33	75	63.03	3.50±1.1	4.16±0.92
60	Blood pressure measurement	6	40.00	57	47.90	-	-
61	Temperature	4	26.67	52	43.70	-	-

62	Body mass index	5	33.33	52	43.70	-	-
63	Fundoscopic examination	4	26.67	72	60.50	-	-
64	Otoscopic examination	4	26.67	69	57.98	-	-
65	Visual acuity	5	33.33	56	47.06	-	-
66	Hearing test with tuning fork	5	33.33	56	47.06	-	-
67	Resting ECG	5	33.33	60	50.42	-	-
68	Peak expiratory flow rate	6	40.00	65	54.62	-	-
69	Glucometer	5	33.33	54	45.38	-	-
70	Pap smear	5	33.33	69	57.98	-	-
71	Nebuliser therapy	5	33.33	72	60.50	-	-
72	Counsel on inhaler technique with and without aerochamber	7	46.67	71	59.66	-	-
73	Writing a prescription	7	46.67	86	72.27	-	-
74	Writing a referral letter	7	46.67	84	70.59	-	-
75	Limitations within primary care	4	26.67	73	61.34	-	-
76	Principles of medical ethics	6	40.00	81	68.07	-	-
77	Behaving professionally in a manner acceptable to the profession and society	5	33.33	69	57.98	-	-
78	Demonstrating the capacity for:<br>Self-awareness, reflection and self-appraisal	5	33.33	63	52.94	-	-
79	Professional development	5	33.33	66	55.46	-	-
80	Lifelong learning	5	33.33	65	54.62	-	-
81	Understanding the personal health risks of medical practice: Fatigue	8	53.33	74	62.18	2.75±0.62	3.93±0.85
82	Understanding the personal health risks of medical practice: Stress	8	53.33	72	60.50	3.00±0.74	4.08±0.85
83	Understanding the personal health risks of medical practice: Occupational hazards	8	53.33	73	61.34	2.83±0.72	4.10±0.76
84	Working effectively and cooperatively as a member of a primary care team	4	26.67	73	61.34	-	-

S2(B) Table: Universiti Putra Malaysia

		Round 1				Round 2	
		Educators		students		Educators	Students
	**Topic**	n	%	n	%	Mean±SD	Mean±SD
1	Honest and integrity	12	48	123	60.0	-	-
2	Accountability and responsibility	13	52	113	55.1	3.91±1.31	3.74±1.03
3	Fitness to practice	8	32	98	47.8	-	-
4	Social Media professionalism	17	68	113	55.1	3.96±1.20	3.95±1.04
5	Doctor patient relationship	17	68	158	77.1	4.00±1.37	4.03±1.11
6	Interprofessional skills (including teamwork)	10	40	118	57.6	-	-
7	Holistic medicine	8	32	134	65.4	3.35±0.93	3.71±1.04
8	Reflective Practice	11	44	95	46.3	-	-
9	Resilience and self care	11	44	88	42.9	-	-
10	Autonomy	7	28	125	61.0	-	-
11	Consent	11	44	142	69.3	3.74±1.24	4.04±1.12
12	Confidentiality	12	48	138	67.3	3.78±1.21	4.05±1.05
13	Basic medical ethics- beneficence, non maleficence, justice	12	48	133	64.9	3.61±0.99	3.75±1.08
14	Ethical reasoning/ decision making	13	52	136	66.3	3.83±1.07	3.98±1.01
15	Assessing capacity	4	16	67	32.7	-	-
16	Truth telling	6	24	106	51.7	-	-
17	Abortion/ euthanasia	7	28	100	48.8	-	-
18	Conflict of interest	11	44	99	48.3	-	-
19	Quality prescribing	7	28	94	45.9	-	-
20	End of life care	12	48	108	52.7	-	-
21	Attentive listening	11	44	126	61.5	-	-
22	Verbal skills and non verbal skills	14	56	143	69.8	3.83±1.23	3.94±1.01

23	Empathy	12	48	125	61.0	-	-
24	Breaking bad news	18	72	170	82.9	3.70±1.23	3.95±1.18
25	Handling angry patient	14	56	145	70.7	3.57±1.15	3.97±1.05
26	Counselling skills	12	48	154	75.1	3.83±1.33	4.10±1.10
27	Written communication (i.e referral letters, medical records)	9	36	130	63.4	-	-
28	Cultural competency and sensitivity	12	48	114	55.6	-	-
29	What is evidence-based medicine	16	64	151	73.7	3.57±1.10	3.50±1.06
30	Asking an answerable question	11	44	125	61.0	-	-
31	Acquire or track down the best evidence	11	44	103	50.2	-	-
32	What is evidence-based practice	14	56	127	62.0	3.57±1.05	3.50±1.13
33	Appraise the evidence I: Primary research	13	52	108	52.7	2.96±1.10	3.43±1.11
34	Appraise the evidence II: Systematic reviews and meta-analysis	15	60	110	53.7	2.96±0.92	3.35±1.18

S2(C) Table: Taylor's University, Pharmacy

		Round 1				Round 2	
		Educators		students		Educators	Students
	**Topic**	n	%	n	%	Mean±SD	Mean±SD
1	Basic properties of cells	1	33.33	22	41.51	4.33±0.58	3.09±1.27
2	Prokaryotic Vs Eukaryotic	2	66.67	16	30.19	4.00±0	3.00±1.16
3	Nucleus	2	66.67	16	30.19	4.00±0	3.09±1.16
4	Endomembrane system	1	33.33	10	18.87	-	-
5	Mitochondria	2	66.67	13	24.53	4.00±0	2.84±1.14
6	Cell surface specialization (cell walls, matrixes, cell junctions)	1	33.33	9	16.98	-	-
7	Cytoskeleton	1	33.33	9	16.98	-	-
8	Cell movements (cilia, flagella, pseudopods)	1	33.33	16	30.19	3.67±1.53	2.93±1.09
9	Cell membrane structure and dynamics	1	33.33	15	28.30	4.00±1.00	3.16±1.10
10	Membrane pumps	3	100.0	10	18.87	2.67±0.58	3.18±1.28
11	Membrane carriers	2	66.67	11	20.75	2.67±0.58	3.18±1.28
12	Membrane channels	3	100.0	8	15.09	2.67±0.58	2.93±1.09
13	Membrane physiology	1	33.33	9	16.98	-	-
14	Chemical nature of gene	1	33.33	15	28.30	3.67±0.58	3.2±1.13
15	Chromosome organization	2	66.67	10	18.87	2.33±1.16	3.00±1.16
16	DNA replication and repair	2	66.67	17	32.08	4.33±0.58	3.48±1.30
17	Heritable structural and chromosome number changes	1	33.33	10	18.87	-	-
18	Gene expression, RNA processing and translation	3	100.00	12	22.64	4.67±0.58	3.55±1.27
19	Cell cycle	1	33.33	20	37.74	4.67±0.58	3.23±1.20
20	Mitosis	3	100.00	21	39.62	4.33±0.58	3.14±1.19
21	Meiosis	3	100.00	21	39.62	4.33±0.58	3.2±1.17
22	Programmed cell death	1	33.33	13	24.53	-	-
23	Plasma membrane receptors	1	33.33	15	28.30	3.33±0.58	3.18±1.28
24	Proteins for signaling	2	66.67	12	22.64	2.33±1.16	3.14±1.23

25	Second messengers	2	66.67	11	20.75	2.33±1.16	2.93±1.09
26	ntegration of signaling	1	33.33	10	18.87	-	-
27	Introduction and overview of microbiology	0	0.00	14	26.42	-	-
28	Difference of prokaryotes and eukaryotes	0	0.00	13	24.53	-	-
29	Classification of microorganisms	2	66.67	15	28.30	3.00±1.00	3.11±1.22
30	Application of microbiology in pharmacy	1	33.33	13	24.53	-	-
31	Microscopic observations of microorganisms and staining techniques	2	66.67	13	24.53	3.00±1.00	3.14±1.23
32	Nomenclature of bacteria	1	33.33	10	18.87	-	-
33	Morphology and fine structures	0	0.00	10	18.87	-	-
34	Nutritional requirements and bacteriological media	0	0.00	10	18.87	-	-
35	Bacteria: Growth and division	3	100.00	16	30.19	3.67±0.58	3.3±1.19
36	Bacterial genetics	1	33.33	14	26.42	-	-
37	Pathogenic, pharmaceutical and beneficial bacteria	0	0.00	10	18.87	-	-
38	Virus: Classification and characteristics	1	33.33	12	22.64	-	-
39	Replication of viruses	2	66.67	13	24.53	3.67±0.58	3.43±1.19
40	Virus inhibition	0	0.00	10	18.87	-	-
41	Control of virus infections, bacterial virus or bacteriophages	1	33.33	10	18.87	-	-
42	Morphology and composition of pathogenic viruses	0	0.00	9	16.98	-	-
43	Pharmaceutical importance of viruses	0	0.00	6	11.32	-	-
44	Fungi and yeasts: Types, morphology	2	66.67	13	24.53	2.67±1.16	3.32±1.10
45	Fungi and yeasts: Reproduction and physiology	1	33.33	13	24.53	-	-
46	Pathogenic yeasts	1	33.33	9	16.98	-	-
47	Pharmaceutical importance of fungi and yeasts	0	0.00	7	13.21	-	-
48	Unicellular and multicellular parasites	1	33.33	14	26.42	-	-
49	Characterization of protozoa, helminths and arthropods	1	33.33	9	16.98	-	-
50	Development of microbial resistance	0	0.00	12	22.64	-	-
51	Carbohydrates: Classification, structure and chemistry	1	33.33	12	22.64	-	-
52	Glycolysis	0	0.00	16	30.19	4.00±1.00	3.57±1.37
53	Tricarboxylic acid cycle, process, controls and inhibitors	0	0.00	15	28.30	3.33±0.58	3.45±1.45

54	Hexose monophosphate pathway	0	0.00	15	28.30	3.33±0.58	3.48±1.37
55	Gluconeogenesis	1	33.33	16	30.19	3.33±0.58	3.61±1.39
56	Glycogenesis	2	66.67	16	30.19	3.33±0.58	3.61±1.39
57	Glycogenolysis	2	66.67	16	30.19	3.33±0.58	3.64±1.38
58	Oxidative phosphorylation	1	33.33	14	26.42	-	-
59	Electron transport chain	1	33.33	16	30.19	4.00±1.00	3.64±1.38
60	Lipids: Classification, structure, chemical characteristics	1	33.33	14	26.42	-	-
61	Lipids: Metabolism	0	0.00	13	24.53	-	-
62	Acid ß-oxidation	2	66.67	16	30.19	2.67±0.58	3.34±1.38
63	Energetic ß-oxidation	2	66.67	14	26.42	2.67±0.58	3.48±1.41
64	Ketone formation	3	100.00	12	22.64	3.00±0	3.41±1.39
65	Biosynthesis of saturated fatty acid	1	33.33	11	20.75	-	-
66	Classification, criteria of amino acids	1	33.33	13	24.53	-	-
67	Essential amino acid	0	0.00	12	22.64	-	-
68	Chemical properties of amino acid	0	0.00	9	16.98	-	-
69	Isoelectrical point of amino acids	0	0.00	9	16.98	-	-
70	Acid-base characteristics of amino acid	0	0.00	9	16.98	-	-
71	Transamination	2	66.67	13	24.53	2.67±0.58	3.41±1.34
72	Oxidative deamination	2	66.67	12	22.64	2.67±0.58	3.14±1.23
73	Decarboxylation	2	66.67	11	20.75	2.67±0.58	3.14±1.23
74	Ketogenic and glucogenic amino acids	2	66.67	12	22.64	2.33±1.16	3.09±1.27
75	Urea cycle	0	0.00	11	20.75	-	-
76	Organisation of protein structure	2	66.67	10	18.87	2.33±1.16	3.09±1.27
77	Properties of proteins	1	33.33	9	16.98	-	-
78	Complex protein	1	33.33	8	15.09	-	-
79	Allosterism	0	0.00	8	15.09	-	-
80	Denaturation of proteins	0	0.00	8	15.09	-	-
81	Enzymes: Classification, nomenclature, basic properties	1	33.33	9	16.98	-	-
82	Enzymes: Substrate specificity	0	0.00	9	16.98	-	-
83	Enzymes: Effects of pH and temperature on enzyme activity	0	0.00	11	20.75	-	-

84	Enzymes: Enzyme denaturation	0	0.00	10	18.87	-	-
85	Enzymes: Enzyme denaturation	0	0.00	9	16.98	-	-
86	Nucleic acid metabolism: De novo synthesis	1	33.33	21	39.62	3.67±0.58	3.43±1.30
87	Nucleic acid metabolism: Degradation	1	33.33	16	30.19	3.67±0.58	3.50±1.29
