# Supplementary material for: Prioritising topics for developing e-learning resources in healthcare curricula: A comparison between students and educators using a modified Delphi survey
Source: PLoS One. 2021 Jun 24;16(6):e0253471. doi: 10.1371/journal.pone.0253471 (PMC8224897; doi:10.1371/journal.pone.0253471)
Supplement: Attachment — Submitted filename: Response to reviewer_PlosOne_31.5.2021.docx [file pone.0253471.s003.docx]

Response to reviewers

Reviewer #1:

The work aim is valid and can be easily replicated in other settings. Nevertheless, there are many confusing points of concern in the manuscript that need to be explained and commented on and, if found necessary to improve the presentation, to make appropriate changes to improve the presented work.

[1] Why in case of TU, 2 different courses (pharmacy and Biomed) were separated in methods and tables but combined only in results? Was there any difference in results figures? Was this a reason to have educator choose completely different choices than students when combined results were presented?

**Response:**

Thank you for highlighting this discrepancy to us. Although it was our original intention to analyse the two courses separately, we did not include TU Biomed data in our final results because only one educator responded in Round 2 which made comparison of responses between students and educators responses unattainable. Therefore, only TU Pharmacy results were reported for TU in Figure 2C and Figure 3C.

We have previously stated it under Methods, line 175-176 "The comparison between students and educators was not done for TU (Biomed) because there was only one educator who responded in Round 2.". We realised that this was not clear and we have revised the manuscript accordingly as follows, under Methods, 2^nd^ paragraph, line 110-114 (*Please refer to the manuscript with tracked changes for the line numbering):*

*"For TU, two courses (Pharmacy and Biomedical) participated in this modified Delphi survey. As the response rate from TU Biomedical was low, where only one educator responded in Round 2 (Round 1: 3 educators, 48 students; Round 2: 1 educator, 40 students), we decided to exclude TU Biomedical from the results and only reported the findings from TU Pharmacy (Supplementary Table S1)."*

[2] In table S2 (B) and figure 3, it is scientifically unacceptable that 25/educator and 205/student agree (independently) to value 16 topics by only 2 values out of the 5-point Likert scale (4 by13/16 and 3 by 3/16)!! This has produced an odd "scatter" plot as shown in figure 3. What went wrong here?

**Response:**

Thank you for pointing out the error in Supplementary Table S2(B) ans Figure 3 and we would like to sincerely apologise for the mistakes. The values were such because the decimal points were rounded up to a whole figure instead of presented in two decimal points like the other institutionas.

We have revised the values to the original two decimal points, and have amended Table S2(B) and figure 3 accordingly. The revised scatterplot shows the spread of the agreement. The revised results did not affect the interpretation and conclusion of this study.

Amended Figure 3B as shown below:

[3] Results, line 152 reads: "topics were sorted in descending order (the most selected to least selected topics) separately for each of student and educator groups. The first quartile of most selected topics from the students and the first quartile of most selected topics from educators were included in Round 2 survey." OK, why item #2 (scoring 52/educator and 55.1/student) was included as a quartile in R2 and not item #23 (scoring 60/educator and 61/student); see (table S2 (B): Supplementary documents). How can the value of 52 and 55.1 be included in a quartile from descending values and in same table values of 60 and 61 (respectively) are not included in the same quartile?? No clue can be discovered.

**Response:**

Thank you for pointing out the discrepancy and we sincerely apologised for the mistake. For item #23, the scoring by educator (n=12) was 48% (not 60%, n=15). The scoring #23 for students was correct i.e. 61% (n=125). Therefore, #23 was not in the first quartile for the educator scoring and hence was not included in Round 2. We have made the amendment in Supplementary material Table S2(B).

[4] Need to comment on the difference in results from TU in comparison with UM and UPM by referring to difference in nature of the lists of "topics". The lists used in UM and UPM cover number of "competencies' while those for TU covered merely listed subjects relating to purely factual knowledge as detailed in the supplemental materials (tables S2 A-D). Accordingly, one major result should emphasise that nature of topics can affect the results. That was also superimposed by the fact that in case of TU there was a large number of topics with a small number of participants especially educators.

**Response:**

Thank you for the suggestions. We have added the following points under Discussion, 4^th^ paragraph, line 260-265 as shown below:

*“Another possible explanation for differences in prioritisation pattern across different universities is the nature of the topics. For instance, TU’s topics were mainly on basic science (knowledge-based) while those of UM and UPM focused on clinical competencies (e.g. prescription and communication). In addition, TU had a large number of topics for selection but only a smaller number of participants, especially educators. This again might contribute to the difference in the prioritization process for TU.”*

[5] There is a need to emphasise that students' role as a participant in identifying their "learning needs" which can improve their outcomes and those of forthcoming batches, are more relevant than role of their educators in identifying their "teaching needs" which can easily be subjective depending on each educator's interest and subspecialty.

**Response:**

We totally agree with the Reviewer and have added the points with supporting references under Discussion, 6^th^ paragraph, line 278-398 to emphasise the relevance of students' role in identifying their own learning needs.

*"Lack of knowledge and understanding among the faculty and educators could be a factor that contributes to an unmet expectation among the students. Hence, it is important to take the students' preference of topics into consideration when developing RLOs. Students have their own experiences what topics that are difficult to understand where RLO would be helpful; or what topics require more multimedia interaction to enhance their learning processes*. *While educators' opinions on the selection of topics for RLO development should be considered as they are the content and education experts, their preference and prioritisation may be influenced by their personal interest and perceptions of the students. Afshar et al. have highlighted how educator’s preferred teaching approach resulted in the loss of interest and reluctance in learning biochemistry among medical students, and how this can be addressed by taking into considerations of the students' learning needs (34). We would, therefore, like to argue that teaching approaches should prioritise students’ needs and preferences over those of the educators. In addition, students' need assessment should be the cornerstone in curriculum planning and development to allow educators in identifying topics, skills and knowledge that address learning needs (35). With needs assessment, learning becomes more relevant to their clinical practice, which is more likely to lead to a change in their practice (36)*."

[6] You need to check values as in table 2, row 1 indicates that (n=) for UPM is 25 and for TU-pharmacy is 3 but the following rows indicate that either the figure in row 1 is reversed or details in following rows are reversed between the two universities.

**Response:**

We apologised for the mistake and have amended the figure accordingly in Table 2.

[7] In figure 2, I suggest using different shapes for the plotted values instead of colours as usually printouts are made in BW rendering the figure to be of less value.

**Response:**

Thank you for your suggestion. We have changed the colour to different shapes and shading as below in Figure 2:

[8] Check text carefully, there are several sentences containing doubled words and misspellings e.g. lines 214, 249.

**Response:**

We have combed through the manuscript and edited all the doubled words accordingly.

[9] Results, line #177 to 178, the sentence states that fig 2 is for students and fell short to mention for educators also.

**Response:**

We have now added the word "educators" in the sentence.

I Suggest rewriting and resubmitting as a study of student learning needs assessment of previous students for future classes' RLO. The needs of the students can give a more valid list than a list of educator's choices of importance of the competencies. Meanwhile, the study should cover competencies in UM and UPM only (exclude factual knowledge of TU).

**Response:**

Thank you for the suggestion to improve the manuscript.

Although we acknowledged that the nature of topics (clinical competencies vs basic science knowledge) might affect the topic selections of students, this study was intended to be exploratory and hope to compare the learning needs of the students with those perceived by the educators across different health disciplines. This, we hope, will identify gaps and findings for future research, particularly whether and how the nature of topics (and other factors) affects students' learning needs.

We address the limitation under Discussion section, last paragraph, line 331-333 as below:

*"The nature of topics was different between institutions where UM's and UPM's topics were related to clinical competencies while TU Pham's topics were basic science knowledge. This might contribute to the discrepancy of results between institutions."*

Reviewer #2:

Very interesting idea - Does the difference in student level at the institutions play a role here?

**Response:**

We agree with the reviewer that the difference in student level at the institutions might influence the results of this present study. We acknowledged this limitation under Discussion, last paragraph, line 334-335 as below:

***"****The students in this study were at different stages of their undergraduate programmes; this might have affected their learning needs and experience. This factor was not further explored in this study."*

I am a bit unclear on your final conclusion. Please consider clarifying the take-away for the reader and perhaps increasing discussion a bit. Overall, very nicely done.

**Response:**

We have refined the conclusion to clarify the take-away message for the readers, under conclusion, line 338-344 as below:

*"This study showed the variations of opinions in topic selection between students and educators across institutions and health topics. Further research is needed to explore the factors influencing the discrepancy in students’ learning needs from the perspective of the students and educators. This study also highlighted the importance of conducting students' learning needs assessment before developing eLearning resources for effective implementation. Learning needs assessment should be the starting point when designing eLearning resources for healthcare curricula."*
